# Bringing evolutionary cancer therapy to the clinic: a systems approach

**DOI:** 10.1038/s41540-025-00528-8

**Published:** 2025-05-27

**Authors:** Arina Soboleva, Irene Grossmann, Anne-Marie C. Dingemans, Jafar Rezaei, Kateřina Staňková

**Affiliations:** 1 Institute for Health Systems Science, Delft University of Technology, Delft, The Netherlands; 2 Department of Pulmonology, Erasmus Medical Center Cancer Institute, Rotterdam, The Netherlands; 3 Faculty of Technology, Policy and Management, Delft University of Technology, Delft, The Netherlands

**Keywords:** Cancer, Computational biology and bioinformatics, Mathematics and computing, Interdisciplinary studies

## Abstract

Evolutionary cancer therapy (ECT) delays or forestalls the progression of metastatic cancer by adjusting treatment based on individual patient and disease characteristics. Clinical implementation of ECT can improve patient outcomes but faces technical and cultural challenges. To address those, we propose a systems approach incorporating systems modeling, problem structuring, and stakeholder engagement. This approach identifies and addresses barriers to implementation, ensuring the feasibility of ECT in clinical practice and enabling better metastatic cancer care.

## Introduction and motivation

Evolutionary cancer therapy (ECT) (also known as adaptive therapy) addresses one of the biggest challenges in cancer treatment, i.e., the rapid development of treatment-induced resistance. ECT typically applies principles of evolutionary game theory (EGT) to forestall this resistance^1–5^. ECT often requires lower treatment doses, decreasing its side effects and improving patients’ quality of life^2,4,6,7^. The approach is patient-specific (treatment protocol depends on the individual disease progression) and adaptive (treatment decisions are based on the disease response)^1,4,5,7,8^. To predict the tumor progression and suggest relevant treatment decisions, ECT uses mathematical models based on known cancer biology from in vitro and in vivo data^3–5,7–9^.

The results of the first clinical study on evolutionary therapy for cancer in 2017 demonstrated a significant increase in time to progression for metastatic castrate-resistant prostate cancer (mCRPC) patients^6^. Since then, a growing number of theoretical studies have focused on the development of cancer dynamics models, validating the models with clinical and experimental data, and designing new ECT protocols (i.e.,^10–13^). Several clinical trials have been initiated in recent years that assess the effectiveness of ECT for different types of cancer and treatments. However, as a model-based treatment innovation, ECT may face difficulties in its implementation in general clinical practice^5,8,14^.

Although the implementation of innovations in healthcare is, in general, challenging^15–17^, the clinical translation of the model-based intervention is also complicated by the limited trust in mathematical models and the difficulties in communication between modelers and healthcare professionals^18–22^. ECT, as a treatment approach that originates from mathematical modeling, is also likely to encounter these barriers. Previous experience shows that when an innovation has an origin outside of the medical world, it is faced with resistance from medical professionals^19,23,24^. Moreover, ECT protocols necessitate careful monitoring of progression, which may demand more resources, making it impractical for routine clinical practice.

Here, we discuss the challenges that the clinical translation of evolutionary cancer therapy faces. We then suggest a systems approach, which uses systems modeling, problem structuring, and iterative stakeholder involvement to address some of the key challenges and facilitate the adoption of ECT in clinical practice.

## Evolutionary cancer therapy

The idea of evolutionary cancer therapy originated around 30 years ago when the principles of evolutionary game theory (EGT) were first applied to the mechanisms of cancer and its treatment^25,26^. EGT for cancer considers games between the different cancer cell populations (for example, treatment-sensitive and treatment-resistant ones), where they compete for resources and space, according to principles of natural selection^1,11,27^, or between cancer and a physician attempting at its control or cure^2–4,28^. In the latter case, the interaction between a physician and the cancer population is viewed as a Stackelberg (or leader-follower) game^4,29,30^. A rational physician (“leader”) makes decisions about the timing and dosing of treatment, while the cancer population (“follower”) can only adapt by developing resistance mechanisms^4,30,31^. The analysis of the game suggests a strategy for the rational physician, which allows the elimination of cancer, postpones its progression, or keeps it under control forever^4,30^.

ECT utilizes mathematical models that describe the dynamics of the cancer population and develops a treatment approach based on the predictions of these models. The most commonly used models are ordinary differential equations (ODEs), partial differential equations (PDEs), and agent-based models (ABMs). The differential equation models typically describe cancer dynamics in response to treatment, measured through a biomarker. PDEs and ABMs elicit the spatial dimension of tumor development, explicit cell interactions in space, and their effects on the development of resistance and effectiveness of the treatment (e.g.,^32,33^).

Before model predictions are applied to the design of an ECT protocol, its adequacy is carefully evaluated. First, the models are calibrated with real-world in vitro and in vivo data and validated in their ability to fit the cancer time-series data in response to treatment (e.g.,^34–36^). The model accuracy depends on the quality of data it is calibrated with. This highlights the importance of uncertainty quantification, which statistically describes measurement error, explores the predictive power of the models against various goodness of fit measures, and evaluates parameter uncertainty^37^. Further, the model predictions and the effectiveness and safety of ECT protocols are evaluated in preclinical in vitro cell culture experiments (e.g.,^10^) and in vivo mice models (e.g.,^1,13^). After careful evaluation, the models are implemented in the design of ECT clinical trial protocols. Moreover, the model used to design a clinical trial can be later recalibrated with the patient data from the same trial, to further improve the model predictions and treatment protocols^7^.

The usual ECT strategies involving one drug are dose skipping (treatment is paused and resumed based on cancer response to the treatment) and dose modulation (the administered treatment dose is adjusted based on the response)^1,5,8,9,13,38^. Multi-drug evolutionary therapies include extinction therapy, which subsequently uses two or more types of drugs to eliminate the cancer population completely, and double bind therapy, which uses two therapies in such a way that the development of the resistance towards one increases susceptibility to another^39–44^.

### The current state of adoption

The first evolutionary therapy clinical trial started in 2015 at Moffitt Cancer Center in Tampa, Florida. The adaptive approach was applied to the treatment of metastatic castrate-resistant prostate cancer (mCRPC)^6,7^. In the study, the patients were treated with a constant dose until the tumor burden (measured as prostate-specific antigen (PSA) level) decreased by 50%. The treatment was then paused to let the tumor regrow to its initial size, after which the treatment was resumed. As a result of the pilot trial, the median time to progression increased to 27 months compared to 16.5 months at the standard of care, while the cumulative drug dose was lowered to 47% of standard dosing^6^. Four years later, the new results of the clinical trial showed the median time to progression of 33.5 months in the adaptive therapy cohort compared to 14.3 months in the standard of care cohort^7^.

The promising results of the initial trial initiated the growth of interest in evolutionary cancer therapy. A number of studies were published on the analysis of the models^12,45,46^, in vitro experiments^11,47–49^, validating models with clinical data and designing new clinical trial protocols^50^. In clinical translation, Moffitt Cancer Center continues to be a pioneer of evolutionary cancer therapy. The fact that Moffitt is a functioning hospital, as well as a research center with the Integrated Mathematical Oncology section, promotes the joint work of modelers and physicians and allows for fast clinical translation of ECT studies. The ongoing trials there apply adaptive protocols for castration-sensitive prostate cancer (NCT03511196; multi-drug therapy NCT05189457), BRAF mutant melanoma (NCT03543969), advanced basal cell carcinoma (NCT05651828), and rhabdomyosarcoma (NCT04388839; both dose adjusting and multi-drug). There is also an evolutionary tumor board feasibility study where a multidisciplinary team suggests evolutionary treatment strategies for incurable patients (NCT04343365)^14^.

Outside the US, there are far fewer evolutionary cancer therapy clinical trials. There is one ongoing trial on adaptive chemotherapy protocol for ovarian cancer in the UK (NCT05080556) and one trial for metastatic castrate-resistant prostate cancer in the Netherlands and Australia (ANZadapt; NCT05393791). Both trials started in 2023 and the results are expected in upcoming years.

## Challenges of clinical translation

Evolutionary cancer therapy requires a multidisciplinary collaboration between medical professionals and researchers in mathematics, evolutionary biology, data science, and statistics. Typically, theoretical researchers need to build collaborations with medical professionals. A few barriers may hinder these collaborations. First, the high workload of medical professionals and the prioritization of tasks of immediate importance over tasks for future development may decrease their ability to be involved in time-demanding research projects^21,51,52^. Another difficulty is the communication barrier between medical professionals and modelers, attributed to their different backgrounds, unfamiliarity with technical language, limited doctors’ experience with mathematical models, and irregular meetings due to doctors’ high workload^18,19,21,22,53^. Moreover, the cultural aspects of the medical field play an important role in the adoption of innovations, and considering these aspects is essential when collaborating with medical professionals^20,21,52,54–56^. For example, the unique power structure of the medical system can have a negative influence on the innovations adoption^21,54–58^. However, it can also be used as leverage for the clinical translation of ECT. If the idea is supported by those high in the medical hierarchy, the chances of it being adopted by others are higher^56,58^. Further, the medical field is typically skeptical about outsider ideas^18,20,56^. It can be a significant barrier to ECT, as it suggests the treatment strategy and thus “interferes” directly with doctors’ domain, which may lead to their distrust and resistance.

In general, lack of trust in models and their predictions is an important consideration in the implementation of model-based innovations in healthcare^18,21,59–61^. The additional difficulty of ECT is that pioneering medical professionals should support the idea before clinical trials can be conducted. In the medical belief system, the trust in innovations is, to a large extent, based on the results of randomized controlled clinical studies^24,59–61^. In the case of ECT, the basis for clinical trials is predictions of the models calibrated with wet lab evidence and results from clinical practice and clinical trials for different types of cancer, which might be perceived as not sufficiently convincing.

The technical barriers to ECT implementation are related to its increased requirements for dynamic follow-up of the disease evolution in response to treatment. The success of ECT is highly dependent on the quality of data, as the tumor dynamics should be carefully followed to determine the next treatment decision^5,8^. While some types of cancer have a reliable biomarker of tumor progression, such as prostate-specific antigen (PSA) in prostate cancer^62,63^, for other types of cancer, more elaborate test methods are needed. For example, CT scans can be used to assess tumor volume. In clinical practice, the first scan is usually performed before the start of the therapy, and the subsequent one is only 6–12 weeks after, and afterwards, scans are repeated once every 3 months^8^. However, for the calibration of ECT models, the tumor measurements around the start of the treatment are critical, as well as more frequent measurements of tumor progression^8,36^. Also, the resolution of the imaging would be important. Commonly used criteria for assessment of tumor response and disease progression, usually RECIST^64^, may not be suitable to determine ECT protocols. In RECIST, only one dimension of the tumor is measured, making the volume estimate imprecise. Also, it is not compatible with ECT protocols that allow tumor burden to regrow, as RECIST directs a stop of the treatment when the tumor increases by more than 20% of its minimum size^64^. The novel, not yet widely applied methods to assess tumor burden, such as the level of circulating tumor DNA (ctDNA) in blood^65–68^, can offer an easier progression follow-up, aiding the ECT approach. Some tumor characteristics (i.e., immuno-suppression, heterogeneity, and adaptability rates) that can inform more accurate ECT models and better treatment decisions are not directly measurable with the current technology^5^. The development of more detailed, personalized, and potentially more effective ECT protocols requires the advancement of cancer testing technologies, which entails significant time and resource investments ^5^.

Looking forward, the effects and consequences of ECT clinical implementation are still to be determined. For instance, more frequent testing can be challenging in some healthcare systems, where the waiting time for testing is already long. Also, some ECT treatment decisions, such as using lower dosages, stopping treatment when it is still effective, and maintaining a high tumor burden to delay the progression, can appear counterintuitive for patients. ECT often involves a trade-off between allowing tumor growth to delay resistance and the associated risks, such as increased mutation rates, the emergence of new metastases, and severe symptoms^8^. These risks raise ethical considerations that need to be carefully evaluated and openly communicated to the patients. Patient acceptance of ECT has not yet been addressed in studies, but we can expect a variety of opinions and different attitudes towards ECT depending on their personal values and treatment goals. For patients to make an informed decision, the approach—along with its risks and benefits—must be thoroughly explained. This would require detailed informed consent and likely longer consultations.

ECT requirements for more frequent tests and longer consultations may raise capacity concerns and increase treatment costs. However, ECT uses less medication^5–7^ and as oncologic drugs are often very expensive, the ECT can lower the medication costs significantly. A small study on the budget impact of ECT for castrate-resistant prostate cancer showed that the mean annual cost of treatment per patient decreased from *$*146, 782 to *$*79, 093, with the largest cut attributed to the reduced drug usage^69^. The financial feasibility of ECT depends on the financing structure of a healthcare system. If the financial burden of the treatment falls on patients, the savings from reduced drug usage should offset the increased costs of additional tests, as financial toxicity is a significant risk factor for medical noncompliance^70^. In healthcare systems with mandatory health insurance, including ECT in the insurance plan is crucial for ensuring patient access to it. Overall, a detailed health economics assessment of the approach is required. Also, depending on the country, the role of regulatory bodies can be significant. For example, in the US, clinical trials are monitored by the Food and Drug Administration (FDA). Thus, communication with them and addressing their requirements for therapy approval is a valuable consideration. Another example is the National Health Care Institute (ZiN) in the Netherlands, which determines the content of the basic insurance package. As we mentioned earlier, insurance coverage of ECT is crucial for its implementation, and the decision of ZiN would have a direct impact on its adoption. To avoid potential stumbling blocks for ECT implementation, we need to ensure the alignment of the key stakeholders’ interests with ECT.

## Systems approach for ECT clinical translation

It is not rare that innovations in healthcare end up not being implemented despite proven technical efficiency^15,16^. It can then require more effort and time on top of what was already invested to demonstrate the benefits of the innovation to the stakeholders, gain support, and ensure the feasibility of its wide use. Although ECT is still being evaluated in clinical trials, we believe that upfront consideration of future implementation is beneficial in the long run.

We propose the systems approach to the clinical translation of ECT. With this approach, we aim to tackle future implementation barriers by considering them in the development of ECT. The goals, which are presented below, directly address the key challenges discussed in the previous section.

1. Foresee the effect of ECT on the healthcare system and ensure its feasibility in clinical practice;
2. Consider different interests of healthcare stakeholders and their influence on the ECT implementation;
3. Establish partnerships with healthcare professionals and build trust.

The approach incorporates systems thinking. In a complex system, interactions of the system’s elements produce an effect that differs from the sum of the individual elements^71,72^. Modern healthcare is a complex system formed by the interplay between various stakeholders with different, potentially conflicting interests^73–77^. The systems approach acknowledges that outcomes of any interventions in healthcare would depend on this interplay and considers it in the development of the intervention^73,77^.

**Fig. 1:**
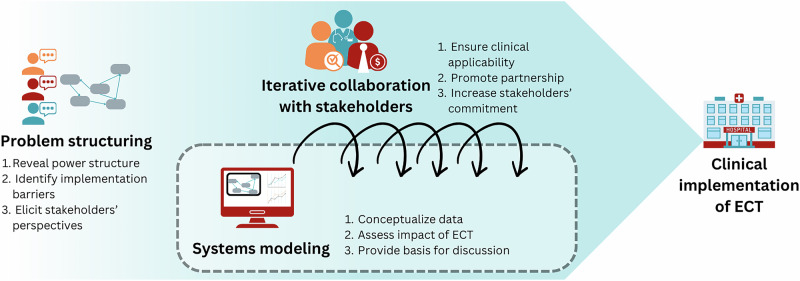
The systems approach to clinical translation of evolutionary cancer therapy. The graph presents three main components of the systems approach: problem structuring, system modeling and collaboration with stakeholders. The goal of the systems approach is to bring evolutionary cancer therapy to clinical implementation.

The workflow of the systems approach is presented in Fig. 1. To begin with, problem structuring methods (PSM), which are used in situations where not only the solution but the problem itself is unknown^78–80^, can uncover the challenges of implementation early on to anticipate them. In the case of ECT, PSM can reveal the attitude of stakeholders, the actual capacity constraints, and other concerns of the practitioners that have not yet been considered. For example, it can explore the requirements to include ECT in insurance plans, physicians’ trust in ECT, and willingness to offer it to their patients, and what frequency of tests is realistic given a hospital’s resource capacity. Additionally, PSM can help identify issues in the medical field that can be addressed by ECT. Carter et al.^56^ suggest that innovation should address a critical issue in healthcare to be successfully implemented. In the case of evolutionary therapy, the critical issue can be the overtreating of patients or the rapid development of resistance to initially effective drugs. If medical professionals recognize these problems, they might be more motivated to engage with ECT as a potential solution.

PSM work directly with the stakeholders through individual interviews or group workshops^78–81^, allowing to learn about the system structure and culture firsthand. When applied in a workshop, PSM also benefits the stakeholders, as they get an opportunity to learn from each other, enrich their own understanding of the system, and improve the collaboration between the organizations in healthcare^78,79,82^. A diverse group of stakeholders, including the end-users, is beneficial as it brings richer insights^83–86^. For ECT, the list of stakeholders can include (but not be limited to) physicians, nurses, health insurers, regulatory bodies, and representatives of patient advocacy groups. PSM results in a structured representation of the problem^78,79,81^. It can reveal the requirements for ECT implementation (e.g., what type of evidence is sufficient, what are the concerns and objectives of patients, and how can the required testing capacity be ensured). After performing the PSM step, these issues can be addressed more effectively with the consideration of stakeholders’ knowledge and interests^78,80,87^.

The next step of the systems approach to ECT clinical translation is to use the systems modeling tools. Such models are focused on the processes at the level of a hospital or healthcare system and often incorporate non-linearity, multiplicity of objectives and delays^88^. First, they can further enhance the learning about the complexity of the healthcare system, providing a formal representation of interconnections, conflicting interests, power relationships, and implicit and explicit stakeholders’ beliefs^82,88–90^. Further, via modeling, the effect of the implementation of ECT on the healthcare system can be estimated. For example, we can hypothesize the frequency of tests needed to inform adaptive protocols and then approximate the burden on the system if the protocol is widely available for the patients. This way, we can assess the feasibility of ECT by testing “what-if” scenarios without resource-intensive investments^89,91^, and provide recommendations on how to adapt it to clinical practice. This is especially valuable considering the scale of the potential effect of evolutionary therapy on the healthcare system and the scarcity of resources in healthcare^20,21^. Finally, modeling helps to establish a “common language” between the stakeholders, elucidate hidden assumptions, add rigor to the group discussion, and can serve as a base for debate^19,92,93^. Such models can aid the negotiations with health insurers, hospitals, and regulatory bodies.

Another important consideration for the future implementation of ECT is the iterative collaboration with the stakeholders, as it is known to benefit the adoption and embedding of innovations^24,56,83,94^. Stakeholder engagement often improves the relevance and quality of research^84,86,95–97^. Medical professionals can bring their experience with real-life cases, which can raise important questions for ECT and challenge its hypothesis. Their involvement can steer evolutionary therapy research into clinically more interesting directions. Besides benefits for the research outcomes, stakeholder engagement also promotes their ownership and commitment to the innovation^84,86,95–97^. Regular meetings allow researchers and healthcare professionals to learn about each other’s worldviews and build trust, therefore improving mutual understanding and narrowing the communication gap^54,58,98^. An illustration of the importance of stakeholder collaboration in the implementation of model-based innovations is the adoption of model-informed precision dosing (MIPD) based on pharmacokinetics and pharmacodynamics^99,100^. It is now implemented in clinical trials to determine effective doses for various oncologic drugs, with some results already implemented in clinic^100^. The gradual acceptance required collaboration between pharmacologists, medical professionals, and regulatory bodies^100,101^. Another example is the joint work of modelers and physicians in Moffitt, facilitated by the shared working environment, which contributed significantly to Moffitt’s pioneering position in the ECT implementation in the clinic. By being exposed to the mathematical models of disease evolution longer, medical professionals can get a better understanding of them and test their explanatory and predictive abilities. Better familiarity leads to increased trust in the models, addressing a key challenge in the adoption of model-informed innovations^52,59,102^. Furthermore, it might encourage them to incorporate disease progression modeling in their work and use it more in the future^83^.

The systems approach to the clinical translation of ECT aims to prepare it to be implemented before the actual implementation stage. It considers the larger system and how different stakeholders might influence the implementation, allowing for the anticipation of potential counteractions. It engages the practitioners early on, ensuring the ECT protocol is well-informed and adjusted to the healthcare system. By the implementation stage, stakeholders are familiar with ECT, thus likely have more trust in it, have established partnerships with the researchers, and are more committed to implementing ECT in clinical practice. This way, the approach addresses some of the key challenges regarding the communication barriers between modelers and medical professionals, trust in the model, and expectations management.

Certainly, the systems approach entails efforts to engage stakeholders, learn about the complex healthcare system, and model the effects of the treatment protocol. The realization of this approach comes with its challenges. The researchers are required to have specific skills in identifying the relevant stakeholders, playing the role of a facilitator, and having a high level of communication skills to manage conflicts^19,84,94^. The modelers should also be able to effectively communicate the mathematical models and their predictions to a non-specialized audience. Naturally, familiarizing medical professionals with modeling tools is not a trivial task. However, given the large potential of mathematical modeling for medical decision support, an approach to educate interested medical professionals should be discussed.

When developing a systems model, the challenging part is mitigating bias and ensuring the model’s validity. As systems models aim to describe real-world phenomena in the healthcare system, quantitative data on the system processes is not sufficient, as it often does not reflect the underlying processes. To ensure that the model is realistic, next to such data, a diverse set of stakeholders should be involved in informing and validating it^52,56,61^. Including perspectives of different stakeholders (not just those at the management levels, but also practitioners) often leads to revealing the system dynamics that were not initially apparent even for the system’s actors^19,83–86^. Therefore, we emphasize the importance of combining the system modeling step with PSM and iterative stakeholder engagement and ensuring a diversity of perspectives to avoid bias. A related concern is the difficulty of motivating the stakeholders to dedicate time to the innovations for which clinical implementation is not yet known^102^. One potential approach is to understand the problems of stakeholders and demonstrate how ECT can help address them. However, suitable incentives for stakeholders and researchers to work together should be further investigated and formulated^103^. Further, the systems approach can lead to delays in the project and higher costs^103–105^. As a payoff, the upfront systems approach can save time and resources at the implementation stage.

## Conclusion

Translation of evolutionary cancer therapy into clinical practice can be challenging and time-consuming. A significant barrier is the lack of trust in the treatment strategy guided by mathematical models. Furthermore, the large scale of the required changes in disease testing and the following challenges, the feasibility of the approach in general clinical practice, necessitates the consideration of the interests of a wide range of stakeholders. We suggest a systems approach to the clinical translation of ECT that addresses the implementation barriers before the implementation stage. The approach investigates and considers stakeholders’ interests, analyzes “what-if” scenarios of ECT adoption, and fosters collaboration between modelers and physicians. The implementation opportunities are investigated at the same time as ECT effectiveness is tested in clinical trials, and not after. Such an approach allows the inclusion of necessary changes in ECT protocols to increase their applicability in clinical practice. Investing more time and effort in stakeholder engagement, identifying potential barriers and solutions, and forecasting outcomes can facilitate the future clinical adoption of ECT, offering improved care opportunities for metastatic cancer patients.

## Data Availability

No datasets were generated or analysed during the current study.

## References

[1] Gatenby, R. A., Silva, A. S., Gillies, R. J. & Frieden, B. R. Adaptive therapy. *Cancer Res.* **69**, 4894–4903 (2009). 19487300 10.1158/0008-5472.CAN-08-3658 PMC3728826

[2] Gatenby, R. A. A change of strategy in the war on cancer. *Nature* **459**, 508–509 (2009). 19478766 10.1038/459508a

[3] Wölfl, B. et al. The contribution of evolutionary game theory to understanding and treating cancer. *Dyn. Games Appl* **12**, 313 (2021). 35601872 10.1007/s13235-021-00397-w PMC9117378

[4] Staňková, K., Brown, J. S., Dalton, W. S. & Gatenby, R. A. Optimizing cancer treatment using game theory: a review. *JAMA Oncol.* **5**, 96 (2019). 30098166 10.1001/jamaoncol.2018.3395 PMC6947530

[5] Strobl, M. A. R., Gallaher, J., Robertson-Tessi, M., West, J. & Anderson, A. R. A. Treatment of evolving cancers will require dynamic decision support. *Ann. Oncol.* **34**, 867–884 (2023). 37777307 10.1016/j.annonc.2023.08.008 PMC10688269

[6] Zhang, J., Cunningham, J. J., Brown, J. S. & Gatenby, R. A. Integrating evolutionary dynamics into treatment of metastatic castrate-resistant prostate cancer. *Nat. Commun.* **8**, 1816 (2017). 29180633 10.1038/s41467-017-01968-5 PMC5703947

[7] Zhang, J., Cunningham, J. J., Brown, J. S. & Gatenby, R. A. Evolution-based mathematical models significantly prolong response to abiraterone in metastatic castrate-resistant prostate cancer and identify strategies to further improve outcomes. *eLife* **11**, e76284 (2022). 35762577 10.7554/eLife.76284 PMC9239688

[8] West, J. et al. A survey of open questions in adaptive therapy: bridging mathematics and clinical translation. *eLife* **12**, e84263 (2023). 36952376 10.7554/eLife.84263 PMC10036119

[9] Cunningham, J. J., Brown, J. S., Gatenby, R. A. & Staňková, K. Optimal control to develop therapeutic strategies for metastatic castrate resistant prostate cancer. *J. Theor. Biol.* **459**, 67–78 (2018). 30243754 10.1016/j.jtbi.2018.09.022

[10] Silva, A. S. et al. Evolutionary approaches to prolong progression-free survival in breast cancer. *Cancer Res.* **72**, 6362–6370 (2012). 23066036 10.1158/0008-5472.CAN-12-2235 PMC3525750

[11] Kaznatcheev, A., Peacock, J., Basanta, D., Marusyk, A. & Scott, J. G. Fibroblasts and alectinib switch the evolutionary games played by non-small cell lung cancer. *Nat. Ecol. Evol.* **3**, 450–456 (2019). 30778184 10.1038/s41559-018-0768-z PMC6467526

[12] Viossat, Y. & Noble, R. A theoretical analysis of tumour containment. *Nat. Ecol. Evol.* **5**, 826–835 (2021). 33846605 10.1038/s41559-021-01428-w PMC8967123

[13] Enriquez-Navas, P. M. et al. Exploiting evolutionary principles to prolong tumor control in preclinical models of breast cancer. *Sci. Transl. Med.* **8**, 327ra24 (2016). 26912903 10.1126/scitranslmed.aad7842 PMC4962860

[14] Robertson-Tessi, M. et al. Feasibility of an evolutionary tumor board for generating novel personalized therapeutic strategies. Preprint at 10.1101/2023.01.18.23284628 (2023).

[15] Mosteller, F. Innovation and evaluation. *Science* **211**, 881–886 (1981). 6781066 10.1126/science.6781066

[16] Morris, Z. S., Wooding, S. & Grant, J. The answer is 17 years, what is the question: understanding time lags in translational research. *J. R. Soc. Med.* **104**, 510–520 (2011). 22179294 10.1258/jrsm.2011.110180 PMC3241518

[17] Dixon-Woods, M., Amalberti, R., Goodman, S., Bergman, B. & Glasziou, P. Problems and promises of innovation: why healthcare needs to rethink its love/hate relationship with the new. *BMJ Qual. Saf.* **20**, i47–i51 (2011). 21450771 10.1136/bmjqs.2010.046227 PMC3066840

[18] Brailsford, S. Overcoming the barriers to implementation of operations research simulation models in healthcare. *Clin. Invest. Med.* **28**, 312–315 (2005). 16450620

[19] Eldabi, T. Implementation issues of modeling healthcare problems: misconceptions and lessons. in: *Proceedings of the 2009 Winter Simulation Conference (WSC)*, pp 1831–1839 (2009).

[20] Tako, A. A. & Robinson, S. Is simulation in health different? *J. Oper. Res. Soc.* **66**, 602–614 (2015).

[21] Jahangirian, M., Taylor, S. J. E., Eatock, J., Stergioulas, L. K. & Taylor, P. M. Causal study of low stakeholder engagement in healthcare simulation projects. *J. Oper. Res. Soc.* **66**, 369–379 (2015).

[22] Scheinker, D. & Brandeau, M. L. Implementing analytics projects in a hospital: successes, failures, and opportunities. *INFORMS J. Appl. Anal.* **50**, 176–189 (2020).

[23] Berwick, D. M. Disseminating innovations in health care. *J. Am. Med. Assoc.* **289**, 1969–1975 (2003). 10.1001/jama.289.15.1969 12697800

[24] Barnett, J., Vasileiou, K., Djemil, F., Brooks, L. & Young, T. Understanding innovators’ experiences of barriers and facilitators in implementation and diffusion of healthcare service innovations: a qualitative study. *BMC Health Serv. Res.* **11**, 342 (2011). 22176739 10.1186/1472-6963-11-342 PMC3265424

[25] Martin, R., Fisher, M., Minchin, R. & Teo, K. Optimal control of tumor size used to maximize survival time when cells are resistant to chemotherapy. *Math. Biosci.* **110**, 201–219 (1992). 1498450 10.1016/0025-5564(92)90038-x

[26] Tomlinson, I. P. M. Game-theory models of interactions between tumour cells. *Eur. J. Cancer* **33**, 1495–1500 (1997). 9337695 10.1016/s0959-8049(97)00170-6

[27] Monro, H. C. & Gaffney, E. A. Modelling chemotherapy resistance in palliation and failed cure. *J. Theor. Biol.* **257**, 292–302 (2009). 19135065 10.1016/j.jtbi.2008.12.006

[28] Hilbe, C., Kleshnina, M. & Staňková, K. Evolutionary games and applications: fifty years of -the logic of animal conflict’. *Dyn. Games Appl.* **13**, 1035–1048 (2023).

[29] Kleshnina, M., Streipert, S., Brown, J. S. & Staňková, K. Game theory for managing evolving systems: challenges and opportunities of including vector-valued strategies and life-history traits. *Dyn. Games Appl.* **13**, 1130–1155 (2023).

[30] Stein, A. et al. Stackelberg evolutionary game theory: how to manage evolving systems. *Philos. Trans. R. Soc. B* **378**, 20210495 (2023). 10.1098/rstb.2021.0495 PMC10024980 36934755

[31] Salvioli, M. et al. Stackelberg evolutionary games of cancer treatment: what treatment strategy to choose if cancer can be stabilized? *Dyn. Games Appl*. 10.1007/s13235-024-00609-z (2024). 10.1007/s13235-024-00609-z PMC12552401 41140641

[32] Gallaher, J. A., Enriquez-Navas, P. M., Luddy, K. A., Gatenby, R. A. & Anderson, A. R. A. Spatial heterogeneity and evolutionary dynamics modulate time to recurrence in continuous and adaptive cancer therapies. *Cancer Res.* **78**, 2127–2139 (2018). 29382708 10.1158/0008-5472.CAN-17-2649 PMC5899666

[33] Strobl, M. A. R. et al. Spatial structure impacts adaptive therapy by shaping intra-tumoral competitions. *Commun. Med.* **2**, 46 (2022). 35603284 10.1038/s43856-022-00110-x PMC9053239

[34] Ghaffari Laleh, N. et al. Classical mathematical models for prediction of response to chemotherapy and immunotherapy. *PLoS Comput. Biol.* **18**, e1009822 (2022). 35120124 10.1371/journal.pcbi.1009822 PMC8903251

[35] Brady-Nicholls, R. et al. Predicting patient-specific response to adaptive therapy in metastatic castration-resistant prostate cancer using prostate-specific antigen dynamics. *Neoplasia* **23**, 851–858 (2021). 34298234 10.1016/j.neo.2021.06.013 PMC8322456

[36] Soboleva, A., Kaznatcheev, A., Cavill, R., Schneider, K. & Staňková, K. Validation of polymorphic Gompertzian model of cancer through in vitro and in vivo data. *PLoS ONE* **20**, e0310844 (2025). 39787141 10.1371/journal.pone.0310844 PMC11717199

[37] Benzekry, S. et al. Classical mathematical models for description and prediction of experimental tumor growth. *PLoS Comput. Biol.* **10**, e1003800 (2014). 25167199 10.1371/journal.pcbi.1003800 PMC4148196

[38] Bayer, P. & West, J. Games and the treatment convexity of cancer. *Dyn. Games Appl.* **13**, 1088–1105 (2023).

[39] Basanta, D., Gatenby, R. A. & Anderson, A. R. A. Exploiting evolution to treat drug resistance: combination therapy and the double bind. *Mol. Pharm.* **9**, 914–921 (2012). 22369188 10.1021/mp200458e PMC3325107

[40] Gatenby, R. A., Artzy-Randrup, Y., Epstein, T., Reed, D. R. & Brown, J. S. Eradicating metastatic cancer and the eco-evolutionary dynamics of anthropocene extinctions. *Cancer Res.* **80**, 613–623 (2020). 31772037 10.1158/0008-5472.CAN-19-1941 PMC7771333

[41] West, J. B. et al. Multidrug cancer therapy in metastatic castrate-resistant prostate cancer: an evolution-based strategy. *Clin. Cancer Res.* **25**, 4413–4421 (2019). 30992299 10.1158/1078-0432.CCR-19-0006 PMC6665681

[42] West, J. et al. Towards multidrug adaptive therapy. *Cancer Res.* **80**, 1578–1589 (2020). 31948939 10.1158/0008-5472.CAN-19-2669 PMC7307613

[43] Luddy, K. A. et al. Evolutionary double-bind treatment using radiotherapy and NK cell-based immunotherapy in prostate cancer. Preprint at 10.1101/2024.03.11.584452 (2024).

[44] Enriquez-Navas, P. M. & Gatenby, R. A. *28—Evolutionary Strategies to Overcome Cancer Cell Resistance to Treatment in Phenotypic Switching.* (Academic Press, 2020) pp 691–703.

[45] Alvarez, F. E. & Viossat, Y. Tumor containment: a more general mathematical analysis. *J. Math. Biol.* **88**, 41 (2024). 38446165 10.1007/s00285-024-02062-3

[46] Kuosmanen, T. et al. Drug-induced resistance evolution necessitates less aggressive treatment. *PLoS Comput. Biol.* **17**, e1009418 (2021). 34555024 10.1371/journal.pcbi.1009418 PMC8491903

[47] Freischel, A. R. et al. Frequency-dependent interactions determine outcome of competition between two breast cancer cell lines. *Sci. Rep.* **11**, 4908 (2021). 33649456 10.1038/s41598-021-84406-3 PMC7921689

[48] Rodriguez Messan, M. et al. Predicting the results of competition between two breast cancer lines grown in 3-D spheroid culture. *Math. Biosci.* **336**, 108575 (2021). 33757835 10.1016/j.mbs.2021.108575

[49] Farrokhian, N. et al. Measuring competitive exclusion in non-small cell lung cancer. *Sci. Adv.* **8**, eabm7212 (2022). 35776787 10.1126/sciadv.abm7212 PMC10883359

[50] Hockings, H. et al. Adaptive therapy achieves long-term control of chemotherapy resistance in high grade ovarian cancer. Preprint at 10.1101/2023.07.21.549688 (2023).

[51] Wilson, J. C. T. Implementation of computer simulation projects in health care. *J. Oper. Res. Soc.* **32**, 825–832 (1981). 10252405 10.1057/jors.1981.161

[52] Brailsford, S. C., Bolt, T., Connell, C., Klein, J. H. & Patel, B. Stakeholder engagement in health care simulation in Proceedings of the 2009 Winter Simulation Conference (WSC), 1840–1849 (IEEE, 2009).

[53] Robinson, S. & Pidd, M. Provider and customer expectations of successful simulation projects. *J. Oper. Res. Soc.* **49**, 200–209 (1998).

[54] do Carmo Caccia-Bava, M., Guimaraes, T. & Harrington, S. J. Hospital organization culture, capacity to innovate and success in technology adoption. *J. Health Organ. Manag.* **20**, 194–217 (2006). 16869354 10.1108/14777260610662735

[55] Brailsford, S. C. *Tutorial: Advances and Challenges in Healthcare Simulation Modeling in 2007 Winter Simulation Conference*. (IEEE, 2007) pp 1436–1448.

[56] Carter, M. W. & Busby, C. R. How can operational research make a real difference in healthcare? Challenges of implementation. *Eur. J. Oper. Res.* **306**, 1059–1068 (2023).

[57] Glouberman, S. & Mintzberg, H. Managing the care of health and the cure of disease—Part I: differentiation. *Health Care Manag. Rev.* **26**, 56 (2001). 10.1097/00004010-200101000-00006 11233354

[58] Bååthe, F. & Erik Norbäck, L. Engaging physicians in organisational improvement work. *J. Health Organ. Manag.* **27**, 479–497 (2013). 24003633 10.1108/JHOM-02-2012-0043

[59] Harper, A., Mustafee, N. & Yearworth, M. The issue of trust and implementation of results in healthcare modeling and simulation studies in 2022 Winter Simulation Conference (WSC), 1104–1115 (2022).

[60] Brailsford, S., Klein, J. H. & Young, T. Evidence from healthcare modeling: what is its nature, and how should it be used? in: *2015 Winter Simulation Conference (WSC)*, 1483–1491 (2015).

[61] Lamé, G., Crowe, S. & Barclay, M. "What’s the evidence?”—towards more empirical evaluations of the impact of OR interventions in healthcare. *Health Systems* **11**, 59–67 (2022). 35127059 10.1080/20476965.2020.1857663 PMC8812794

[62] Carvalhal, G. F. et al. Correlation between serum PSA and cancer volume in prostate glands of different sizes. *Urology* **76**, 1072–1076 (2010). 20846711 10.1016/j.urology.2009.11.056 PMC2975771

[63] Mandel, P. et al. Influence of tumor burden on serum prostate-specific antigen in prostate cancer patients undergoing radical prostatectomy. *Front. Oncol.* **11**, 656444 (2021). 34395240 10.3389/fonc.2021.656444 PMC8358926

[64] Eisenhauer, E. et al. New response evaluation criteria in solid tumours: revised RECIST guideline (version 1.1). *Eur. J. Cancer* **45**, 228–247 (2009). 19097774 10.1016/j.ejca.2008.10.026

[65] Gouda, M. A. et al. Liquid biopsy response evaluation criteria in solid tumors (LB-RECIST). *Ann. Oncol.* **35**, 267–275 (2024). 38145866 10.1016/j.annonc.2023.12.007

[66] Krishnamurthy, N., Spencer, E., Torkamani, A. & Nicholson, L. Liquid biopsies for cancer: coming to a patient near you. *J. Clin. Med.* **6**, 3 (2017). 28054963 10.3390/jcm6010003 PMC5294956

[67] Yee, S. S. et al. A novel approach for next-generation sequencing of circulating tumor cells. *Mol. Genet.* **4**, 395–406 (2016). 10.1002/mgg3.210 PMC4947859 27468416

[68] Di Meo, A., Bartlett, J., Cheng, Y., Pasic, M. D. & Yousef, G. M. Liquid biopsy: a step forward towards precision medicine in urologic malignancies. *Mol. Cancer* **16**, 80 (2017). 28410618 10.1186/s12943-017-0644-5 PMC5391592

[69] Mason, N. T. et al. Budget impact of adaptive abiraterone therapy for castration-resistant prostate cancer. *Am. Health Drug Benefits* **14**, 15–20 (2021). 33841621 PMC8025923

[70] Knight, T. G. et al. Financial toxicity intervention improves outcomes in patients with hematologic malignancy. *JCO Oncol Pract.* **18**, e1494–e1504 (2022). 35709421 10.1200/OP.22.00056

[71] Maier, M. W. & Rechtin, E. *The Art of Systems Architecting* (CRC Press, 2002), 2nd ed.

[72] Sterman, J. D. Learning from evidence in a complex world. *Am. J. Public Health* **96**, 505 (2006). 16449579 10.2105/AJPH.2005.066043 PMC1470513

[73] Savigny, D. D., Adam, T. *Systems Thinking for Health Systems Strengthening* (World Health Organization, 2009).

[74] Mingers, J. & White, L. A review of the recent contribution of systems thinking to operational research and management science. *Eur. J. Oper. Res.* **207**, 1147–1161 (2010).

[75] Atun, R. Health systems, systems thinking and innovation. *Health Policy Plan.* **27**, iv4–iv8 (2012). 23014152 10.1093/heapol/czs088

[76] Rusoja, E. et al. Thinking about complexity in health: a systematic review of the key systems thinking and complexity ideas in health. *J. Eval. Clin. Pract.* **24**, 600–606 (2018). 29380477 10.1111/jep.12856

[77] Komashie, A. et al. Systems approach to health service design, delivery and improvement: a systematic review and meta-analysis. *BMJ Open* **11**, e037667 (2021). 33468455 10.1136/bmjopen-2020-037667 PMC7817809

[78] Mingers, J. & Rosenhead, J. (eds.) *Rational Analysis for A Problematic World Revisited: Problem Structuring Methods for Complexity, Uncertainty and Conflict* (Wiley, 2001), 2nd ed.

[79] Smith, C. M. & Shaw, D. The characteristics of problem structuring methods: a literature review. *Eur. J. Oper. Res.* **274**, 403–416 (2019).

[80] Gomes Júnior, A. D. A. & Schramm, V. B. Problem structuring methods: a review of advances over the last decade. *Syst. Pract. Action Res.* **35**, 55–88 (2022).

[81] Abuabara, L. & Paucar-Caceres, A. Surveying applications of strategic options development and analysis (SODA) from 1989 to 2018. *Eur. J. Oper. Res.* **292**, 1051–1065 (2021).

[82] Voinov, A. et al. Tools and methods in participatory modeling: selecting the right tool for the job. *Environ. Model. Softw.* **109**, 232–255 (2018).

[83] Kumar, M. & Vinati, V. Stakeholder engagement and collaboration in health policy implementation: lessons learned. South East. *Eur. J. Public Health* **23**, 104–119 (2024).

[84] Selby, J. V. & Slutsky, J. R. Practicing partnered research. *J. Gen. Intern. Med.* **29**, 814–816 (2014). 25355093 10.1007/s11606-014-3046-z PMC4239288

[85] Leviton, L. C. & Melichar, L. Balancing stakeholder needs in the evaluation of healthcare quality improvement. *BMJ Qual. Saf.* **25**, 803–807 (2016). 26893512 10.1136/bmjqs-2015-004814 PMC5050280

[86] Grindell, C., Coates, E., Croot, L. & O’Cathain, A. The use of co-production, co-design and co-creation to mobilise knowledge in the management of health conditions: a systematic review. *BMC Health Serv. Res.* **22**, 877 (2022). 35799251 10.1186/s12913-022-08079-y PMC9264579

[87] Marttunen, M., Lienert, J. & Belton, V. Structuring problems for multi-criteria decision analysis in practice: a literature review of method combinations. *Eur. J. Oper. Res.* **263**, 1–17 (2017).

[88] Khalil, H. & Lakhani, A. Using systems thinking methodologies to address health care complexities and evidence implementation. *JBI Evid. Implement.* **20**, 3 (2022). 10.1097/XEB.0000000000000303 34845166

[89] van der Leeuw, S. E. Why model? *Cybern. Syst.* **35**, 117–128 (2004).

[90] Jun, G. T. et al. Development of modelling method selection tool for health services management: from problem structuring methods to modelling and simulation methods. *BMC Health Serv. Res.* **11**, 108 (2011). 21595946 10.1186/1472-6963-11-108 PMC3130639

[91] Pitt, M., Monks, T., Crowe, S. & Vasilakis, C. Systems modelling and simulation in health service design, delivery and decision making. *BMJ Qual. Saf.* **25**, 38–45 (2016). 26115667 10.1136/bmjqs-2015-004430

[92] Vennix, J. A. M. Group model-building: tackling messy problems. *Syst. Dyn. Rev.* **15**, 379–401 (1999).

[93] Monks, T. Operational research as implementation science: definitions, challenges and research priorities. *Implement. Sci.* **11**, 81 (2016). 27268021 10.1186/s13012-016-0444-0 PMC4895878

[94] Petkovic, J. et al. Key issues for stakeholder engagement in the development of health and healthcare guidelines. *Res. Involv. Engagem.* **9**, 27 (2023). 37118762 10.1186/s40900-023-00433-6 PMC10142244

[95] Jagosh, J. et al. Uncovering the benefits of participatory research: implications of a realist review for health research and practice. *Milbank Q* **90**, 311–346 (2012). 22709390 10.1111/j.1468-0009.2012.00665.x PMC3460206

[96] Schmittdiel, J. A., Grumbach, K. & Selby, J. V. System-based participatory research in health care: an approach for sustainable translational research and quality improvement. *Ann. Fam. Med.* **8**, 256–259 (2010). 20458110 10.1370/afm.1117 PMC2866724

[97] Sheldrick, R., Cruden, G., Schaefer, A. & Mackie, T. Rapid-cycle systems modeling to support evidence-informed decision-making during system-wide implementation. *Implement. Sci. Commun.* **2**, 116 (2021). 34627399 10.1186/s43058-021-00218-6 PMC8502394

[98] Linnéusson, G., Andersson, T., Kjellsdotter, A. & Holmén, M. Using systems thinking to increase understanding of the innovation system of healthcare organisations. *J. Health Organ. Manag.* **36**, 179–195 (2022). 35788441 10.1108/JHOM-01-2022-0004 PMC9897203

[99] Jimenez, R. B. et al. Oncologists’ perspectives on individualizing dose selection for patients with metastatic cancer. *JCO Oncol Pract* **18**, e1807–e1817 (2022). 36126244 10.1200/OP.22.00427

[100] Agema, B. C., Koch, B. C. P., Mathijssen, R. H. J. & Koolen, S. L. W. From prospective evaluation to practice: model-informed dose optimization in oncology. *Drugs* **85**, 487–503 (2025). 39939511 10.1007/s40265-025-02152-6 PMC11946950

[101] Darwich, A., Ogungbenro, K., Hatley, O. & Rostami-Hodjegan, A. Role of pharmacokinetic modeling and simulation in precision dosing of anticancer drugs. *Transl. Cancer Res.* **6**, S1512–S1529 (2017).

[102] Harper, A. & Mustafee, N. Participatory design research for the development of real-time simulation models in healthcare. *Health Systems* **12**, 375–386 (2023). 38235299 10.1080/20476965.2023.2175730 PMC10791085

[103] Bowen, S. J. & Graham, I. D. From knowledge translation to engaged scholarship: promoting research relevance and utilization. *Arch. Phys. Med. Rehabil.* **94**, S3–S8 (2013). 23141502 10.1016/j.apmr.2012.04.037

[104] Maurer, M. et al. Understanding the influence and impact of stakeholder engagement in patient-centered outcomes research: a qualitative study. *J Gen Intern Med* **37**, 6–13 (2022). 35349017 10.1007/s11606-021-07104-w PMC8993962

[105] Bartunek, J., Trullen, J., Bonet, E. & Sauquet, A. Sharing and expanding academic and practitioner knowledge in health care. *J. Health Serv. Res. Policy* **8**, 62–68 (2003). 14596750 10.1258/135581903322405199

